# Differences in perception of the WHO International Code of Marketing of Breast Milk Substitutes between pediatricians and obstetricians in Japan

**DOI:** 10.1186/1746-4358-1-12

**Published:** 2006-08-22

**Authors:** Katsumi Mizuno, Fumihiro Miura, Kazuo Itabashi, Iona Macnab, Noriko Mizuno

**Affiliations:** 1 Showa University of Medicine, Department of Pediatrics, Tokyo, Japan

## Abstract

**Background:**

The World Health Organization International Code of Marketing of Breast-Milk Substitutes (WHO Code) aims to protect and promote breastfeeding. Japan ratified the WHO Code in 1994, but most hospitals in Japan continue to receive free supplies of infant formula and distribute discharge packs to new mothers provided by infant formula companies. The aim of this study was to explore the knowledge and attitudes of pediatricians and obstetricians in Japan to the WHO Code.

**Methods:**

A self-completion questionnaire was sent to 132 pediatricians in the 131 NICUs which belonged to the Neonatal Network of Japan, and to 96 chief obstetricians in the general hospitals in the Kanto area of Japan, in 2004.

**Results:**

Responses were received from 68% of pediatricians and 64% of obstetricians. Sixty-six percent of pediatricians agreed that "Breastmilk is the best", compared to only 13% of obstetricians. Likewise, pediatricians were more likely to be familiar with the WHO Code (51%) than obstetricians (18%).

**Conclusion:**

In Japan, pediatricians and obstetricians, in general, have low levels of support for breastfeeding and low levels of familiarity with the WHO Code. To increase the breastfeeding rates in Japan, both pediatricians and obstetricians need increased knowledge about current infant feeding practices and increased awareness of international policies to promote breastfeeding.

## Background

The WHO International Code of Marketing of Breast-Milk Substitutes (WHO Code) aims to protect and promote breastfeeding, and ensure the proper use of breast milk substitutes (Table 1) [1,2]. Japan ratified the WHO Code in 1994, but there are no penal regulations for the Code in Japan.

**Table 1:** International Code of Marketing of Breast Milk Substitutes

Summary of the Code:
1. There should be no advertising of breast milk substitutes or other form of promotion to the general public.
2. Manufacturers and distributors should not provide, directly or indirectly, to pregnant women, mother or members of their families, samples of their products, including discount coupons.
3. No promotion of products in health care facilities.
4. No sales representatives to advise mothers.
5. No gifts or personal samples to health workers.
6. No words or pictures idealizing artificial feeding, including pictures of infants on the labels of the products.
7. Information to health workers should be scientific and factual.
8. All information on artificial infant feeding, including the labels, should explain the benefits of breastfeeding, and the costs and hazards associated with artificial feeding.
9. Unsuitable products, such as sweetened condensed milk, should not be promoted for babies.
All products should be of a high quality and take account of the climatic and storage conditions of the country where they are used.

It is known anecdotally that most hospitals in Japan receive free supplies of infant formula or at significant discount in exchange for distributing hospital-discharge packs prepared by infant formula companies to new mothers. Many hospitals also receive other support from infant formula companies: equipment, educational grants and support for social activities [3]. The close relationship between manufacturers of infant formula and hospitals ensures that new mothers have ready access to infant formula leading to unnecessary supplementation with formula, and eroding support for breastfeeding.

In 2000, the full breastfeeding rate at 1–2 months of age in Japan was 44.8% [4]. A cross-sectional study of 15,262 infants aged three to six months (2001–2004) in Nishinomiya City, Japan, found that 43.8% of infants were fed breast milk only, while 30.4% received infant formula in addition to breast milk [5]. These breastfeeding rates are lower than in the USA (54.7% at 1 month and 47% at 3months, [6]), Australia (64.3% at 13 weeks and 49.0% at 25 weeks [7]) and Sweden (80.2% at 2 months [8]). Hofvander links the increase in breastfeeding rates in Sweden with the Baby Friendly Hospital Initiative, which presupposes compliance with the WHO Code [8].

In Japan, not only pediatricians, but also obstetricians actively participate in infant nutrition decision-making in general hospitals Therefore, both pediatricians and obstetricians need to be aware of the WHO Code.

The aim of this questionnaire survey was to ascertain the level to which awareness and understanding of the WHO Code has penetrated into Japanese pediatric and obstetric practice. Armed with this knowledge, we can more effectively plan the action needed to be taken in order to increase breastfeeding rates in Japan.

## Methods

A self-completion questionnaire was sent to 132 pediatricians in the 131 NICUs which belonged to the Japanese Neonatal Network, and to 96 chief obstetricians who belonged to Obstetric Kanto Network in Japan in 2004. The questionnaire was sent via email and participants were asked to return the completed form by fax, to maintain anonymity. The response rate was 68% (90/132) from the pediatricians and 64% (62/96) from the obstetricians. Chi-square tests were used to compare the groups using Epi-Info [9].

The questionnaire (see Additional file 1) was developed by a group of International Board Certified Lactation Consultants (IBCLCs) in Japan, and circulated to all IBCLCs in Japan (n = 48) for comment to ensure content validity.

## Results

**Figure 1:**
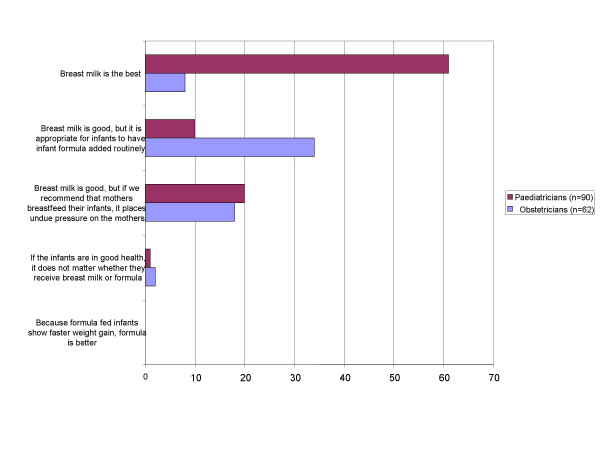
Appropriate neonatal nutrition: responses from pediatricians and obstetricians

Figure 1 presents the response of participants when asked to choose the statement that "most resembles your ideas of appropriate infant nutrition". Pediatricians were more likely to answer in favour of breast milk (chi-square = 50.30, p < 0.01). Sixty-six percent of pediatricians (61/90) answered "Breast is best", with "Breast milk is good, but if we recommend that mothers breastfeed their infants, it places undue pressure on the mothers" the second most frequent response (22%, 10/90). In contrast, 55% (34/62) of obstetricians chose "Breast milk is good, but it is appropriate for infants to have infant formula added routinely" and the second most frequent response (29%, 18/62) was "Breast milk is good, but if we recommend that mothers breastfeed their infants, it places undue pressure on the mothers". Only 13% (8/62) of obstetricians chose "Breast is best".

Responses to this question were stratified by participants' age. Older pediatricians were more likely to chose "Breast is best" than younger ones: 79% (33/42) aged over 50 years compared to 50% (5/10) younger than 40 years, however the difference was not statistically significant (chi-square = 3.35, p = 0.07). Among the obstetricians there was no difference between older and younger doctors.

Participants were asked to describe the infant nutrition that they order for their patients. Only 12% (11/90) of pediatricians and 3% (2/62) of obstetricians routinely ordered "breast milk only". The majority of pediatricians (78%, 70/90) ordered "breast milk, and if it is not enough, add infant formula". Sixty-six percent (41/62) of obstetricians ordered "just milk". Twenty-two percent (20/90) of pediatricians and 34 % (27/62) of obstetricians replied that infant feeding consultations were given by dietitians from infant formula companies.

Among the obstetricians, only 18% (12/62) have heard of the WHO Code, whereas 51% (46/90) of pediatricians "had heard of the Code" or "knew it well".

The doctors who had at least heard of the existence of the Code (46 pediatricians and 12 obstetricians) responded to further questions about the Code. Doctors who "knew the Code well" or "had heard of it", acknowledged that the advertisement of infant formula, infant feeding bottles and artificial teats were prohibited by the Code. Only 22 pediatricians (and no obstetricians) responded correctly to the five true/false items about the WHO Code. The number of doctors who made less than two mistakes was 37 pediatricians (78%) and only 4 obstetricians (33%). Pediatricians who made less than two mistakes were more likely to have selected "breastmilk is best" (32/37) than pediatricians who were not aware of the Code or made 2–3 mistakes (chi-square = 10.7, p = 0.001).

Sixteen of the 46 pediatricians replied that their hospital complies with the WHO Code (35%). Only three of the 18 obstetricians replied that their hospital complies with the Code (17%). Although 80% (24/30) pediatricians think their hospitals should comply with the Code in future, only 39% (7/18) of obstetricians think this. In terms of future likelihood of complying with the Code, more than half of the obstetricians picked "contract with formula company" as the issue which would need to be altered in order for their hospital to comply with the Code. All the obstetricians agreed that obstetricians need to change their attitude to breastfeeding. In terms of pediatricians, 17/28 (61%) pointed out that obstetricians need to change their attitude and about half of pediatricians thought that pediatricians, midwives, nurses and the administrator of the hospital need to change their attitudes and to change the contract with the formula company.

## Discussion

This questionnaire survey elucidated that more pediatricians than obstetricians in Japan considered breast milk the appropriate choice for neonatal nutrition. In addition, it appeared that older pediatricians were more supportive of breastfeeding than younger doctors. Pediatricians who knew the WHO Code in detail were more likely to consider breast milk the best nutrition than doctors less familiar with the Code. In other words, it is important to know in detail about the Code and just knowing the name of the Code does not affect infant feeding choice.

Pediatricians who work in the NICUs have more knowledge of neonatal nutrition, so that they choose breast milk as the best nutrition. Obstetricians, being less familiar with neonatal nutrition, are more likely to consider infant formula as satisfactory nourishment for the neonate.

One of the issues we have to change in Japan is the making of contracts with formula companies. Between 20% and 30 % of general hospitals ask formula companies to send dietitians for infant feeding consultations with mothers. Health care providers, who have heard of the WHO Code, knew or assumed that infant formula, infant feeding bottles, and artificial teats are included in the scope of the Code.

In order to be accredited as a Baby Friendly Hospital, it is necessary to practice all of the Ten Steps for Successful Breastfeeding [8] as well as comply with the WHO Code. Currently, there are 40 Baby Friendly Hospitals in Japan and the exclusive breastfeeding rates in these hospitals are 97–99% at discharge from the maternity ward [4]. It is not known how the breastfeeding rate in Japan would be increased by compliance with the WHO Code alone, but pediatricians who have correct knowledge about the Code consider breast milk the best nutrition. In addition, obstetricians need correct knowledge about infant nutrition and to eliminate contracts with infant formula companies in order to substantially increase breastfeeding rates in Japan. Although older pediatricians understand the importance of breastfeeding and consider breast milk the best choice for the neonate, younger pediatricians and obstetricians are not aware of the importance of breastfeeding. Therefore, there is an urgent need to educate medical professionals as well as future medical professionals.

Even many of the obstetricians who knew of the WHO Code did not think their hospitals should comply with the Code in the future.

## Conclusion

In Japan, more pediatricians are aware of the importance of the WHO Code compared to obstetricians. In addition, most older pediatricians and pediatricians who know the code in detail consider "breast milk is the best" nutrition for infants. We need to educate residents and medical students, not only about the value of breast milk, but also about the Code and have obstetricians comply with the Code in order for mothers to keep breastfeeding their infants.

## Competing interests

The author(s) declare that they have no competing interests.

## Authors' contributions

Dr. Mizuno and Ms. Mizuno IBCLC participated in development of the questionnaire form and drafting the manuscript.

Dr. Miura participated in sending out the questionnaire and analysis of the answers.

Dr. Itabashi participated in analysis of the answers.

Ms. Macnab IBCLC participated in writing the manuscript.

## Supplementary Material

Additional file 1 International Code of Marketing of Breast Milk Substitutes Click here for file
