# Supplementary material for: Differences in perception of the WHO International Code of Marketing of Breast Milk Substitutes between pediatricians and obstetricians in Japan
Source: Int Breastfeed J. 2006 Aug 22;1:12. doi: 10.1186/1746-4358-1-12 (PMC1560113; doi:10.1186/1746-4358-1-12)
Supplement: Additional file 1 — International Code of Marketing of Breast Milk Substitutes [file 1746-4358-1-12-S1.doc]

**International Code of Marketing of Breastmilk Substitutes**

**Below is a summary of the Code:**

- **There should be no advertising of breastmilk substitutes or other form of promotion to the general public.**
- **Manufacturers and distributors should not provide, directly or indirectly, to pregnant women, mother or members of their families, samples of their products, including discount coupons.**
- **No promotion of products in health care facilities.**
- **No sales representatives to advise mothers.**
- **No gifts or personal samples to health workers.**
- **No words or pictures idealizing artificial feeding, including pictures of infants on the labels of the products.**
- **Information to health workers should be scientific and factual.**
- **All information on artificial infant feeding, including the labels, should explain the benefits of breastfeeding, and the costs and hazards associated with artificial feeding.**
- **Unsuitable products, such as sweetened condensed milk, should not be promoted for babies.**
- **All products should be of a high quality and take account of the climatic and storage conditions of the country where they are used.**

**Media Watch Kit**

**To report positive or negative images of breastfeeding in the media.**

**Texas Department of Health
WIC--Breastfeeding Promotion Section
1100 W. 49th Street512-406-0744
Austin, TX 78756**

***The International Code of Marketing of Breastmilk Substitutes: A Common Review and Evaluation Framework*
a document containing the Code, questionnaires, and a discussion of the issues**

**WHO Publications Centre, USA
49 Sheridan Avenue
Albany, NY 12210 518-436-9686**
